# Raynaud’s phenomenon and positive antinuclear antibodies as first manifestation of POEMS syndrome (polyneuropathy, organomegaly, endocrinopathy, monoclonal gammopathy, and skin changes): a case report

**DOI:** 10.1186/s41927-022-00258-y

**Published:** 2022-05-19

**Authors:** Fabio Torres-Saavedra, Lina León-Sierra

**Affiliations:** 1 Division of Rheumatology, GRUA Investigation Group, Universidad de Antioquia, 050010 Medellín, Colombia; 2 IPS Artmedica, Medellín, Colombia; 3 Internal Medicine, Universidad Nacional de Colombia, Bogotá, Colombia

**Keywords:** Raynaud disease, POEMS syndrome, Paraneoplastic polyneuropathy, Autoimmune disease, Case report

## Abstract

**Background:**

POEMS syndrome is a rare paraneoplastic syndrome caused by plasma cell disorder almost always lambda restricted. Secondary Raynaud’s phenomenon is an overlooked skin manifestation of the disease even though it is present in twenty percent of patients. On POEMS syndrome have not been described positive antinuclear antibodies (ANA) and this could lead to a misdiagnosis of autoimmune disease, mainly systemic sclerosis.

**Case presentation:**

A 47-year-old man presented with color changes on fingertips consistent with biphasic Raynaud’s phenomenon; an antinuclear antibody test was positive (at 1:320 titers in a speckled pattern) with normal nailfold capillaroscopy. Clinical features of systemic sclerosis were absent. Twenty-four months later, the patient presented symmetric sensorimotor demyelinating polyneuropathy, and he was diagnosed with Guillain–Barre syndrome; treatment with intravenous gammaglobulin had an incomplete response. Raynaud’s phenomenon persisted associated with acrocyanosis, white nails, and positive ANA (1:1280 in a nucleolar pattern). POEMS syndrome was suspected, and serum protein electrophoresis (SPEP) was done. The SPEP revealed polyclonal gammopathy, and serum immunofixation showed monoclonal (M)-protein (IgG lambda). Serum vascular endothelial growth factor concentration showed increased levels. The patient was diagnosed with POEMS syndrome, and treatment with lenalidomide and dexamethasone improved the Raynaud’s phenomenon, acrocyanosis, and white nails, but the neurological response was partial.

**Conclusions:**

POEMS syndrome may mimic clinical manifestations of systemic sclerosis v.g. Raynaud’s phenomenon, skin thickening, telangiectasia, and positive ANA. Raynaud’s phenomenon may precede other clinical manifestations of POEMS syndrome by several months. It is necessary to have a high index of suspicion for the diagnosis, especially in patients with peripheral polyneuropathy and monoclonal paraprotein. The significance of positive ANA in this condition is unknown and deserves further investigation.

## Background

POEMS syndrome is a rare disease described for the first time in 1938. In 1980, the acronym “POEMS” was suggested to describe the clinical features of this disease (Polyneuropathy, Organomegaly, Endocrinopathy, Monoclonal protein, Skin changes).

In recent years POEMS syndrome has been widely recognized. However, prevalence studies are scarce. A nationwide epidemiologic survey in Japan estimated a prevalence of 0.3 per 100.000 and is considered less in western countries [1].

This disorder is defined by the presence of peripheral neuropathy and a monoclonal plasma cell disorder (IgA/IgG lambda restricted) [2]. Other manifestations are Castleman’s disease, sclerotic bone lesions, organomegaly, extravascular volume overload, increased cerebrospinal fluid protein, endocrinopathy, skin changes, papilledema, thrombocytosis, polycythemia, thromboembolic disease, and elevated VEGF levels.

The cause of the syndrome is unknown, although increased levels of proinflammatory and proangiogenic cytokines, including interleukin-1 beta (IL-1β), tumor necrosis factor-alpha (TNF-α), interleukin-6 (IL-6), and VEGF have been reported [3].

The prevalence of cutaneous manifestations in POEMS syndrome is approximately 68–90 percent [4, 5]. Skin changes in POEMS syndrome are overlooked, in a retrospective study from Mayo Clinic, the mean number of skin findings per patient was 2.9 (median 3; range 0–7) (Table 1) [5]. Raynaud’s phenomenon frequently encompasses other manifestations of POEMS syndrome and is an important feature of the disease. A significant association exists between vascular skin changes and abnormal pulmonary function tests [5].

**Table 1:** Prevalence of cutaneous manifestations in POEMS syndrome [4]

Feature	Patient (%)
Hyperpigmentation	50 (47)
Hemangioma	50 (47)
Hypertrichosis	41 (38)
Acrocyanosis	36 (34)
White nails	32 (30)
Sclerodermoid changes	28 (26)
Raynaud’s Phenomenon	21 (20)
Hyperemia/erythema	21 (20)
Flushing	17 (16)
Rubor	12 (11)
Clubbing	6 (6)

For that reason, secondary Raynaud’s phenomenon is a relevant clue for POEMS syndrome diagnosis and is necessary to establish a rational approach with other diseases that present Raynaud’s phenomenon, mainly autoimmune diseases. We report a case of POEMS syndrome whose first manifestations were Raynaud’s phenomenon and positive antinuclear antibodies.

## Case presentation

A 47-year-old man presented to the clinic reporting color changes on fingertips, initially pale fingers, followed by blue discoloration and numbness. He denied photosensitivity, oral ulcers, skin changes, cough, dyspnea, gastrointestinal symptoms, or urinary changes. Medical history was remarkable for recurrent superficial venous thrombosis of the left leg treated with rivaroxaban.

Physical examination revealed pale fingers with hypertrichosis without puffy fingers, sclerodactyly, telangiectasia, digital ulcers, or calcinosis cutis. No hepatosplenomegaly or lymphadenopathy was present. Initial laboratory studies showed elevated platelets (479 × 10^9^/L, reference value 150–450) with normal hemoglobin concentration (15.3 g/dL, reference value 11.5–15.5), hematocrit levels (44%, reference value 41–50), acute phase reactants (ESR and CRP), thyroid-stimulating hormone (TSH), clotting profile (PT and TPT), creatinine, and B12 vitamin levels. ANA were positive at 1:320 titers in a speckled pattern. Tests for extractable nuclear antigen (ENA), anti-dsDNA titers, complement, antiphospholipid antibodies, cryoglobulins, antineutrophil cytoplasmic antibody (ANCA) by direct immunofluorescence, and nailfold capillaroscopy were unremarkable. Therefore, a diagnosis of secondary Raynaud’s phenomenon was made, treatment with acetylsalicylic acid was initiated.

**Fig. 1:**
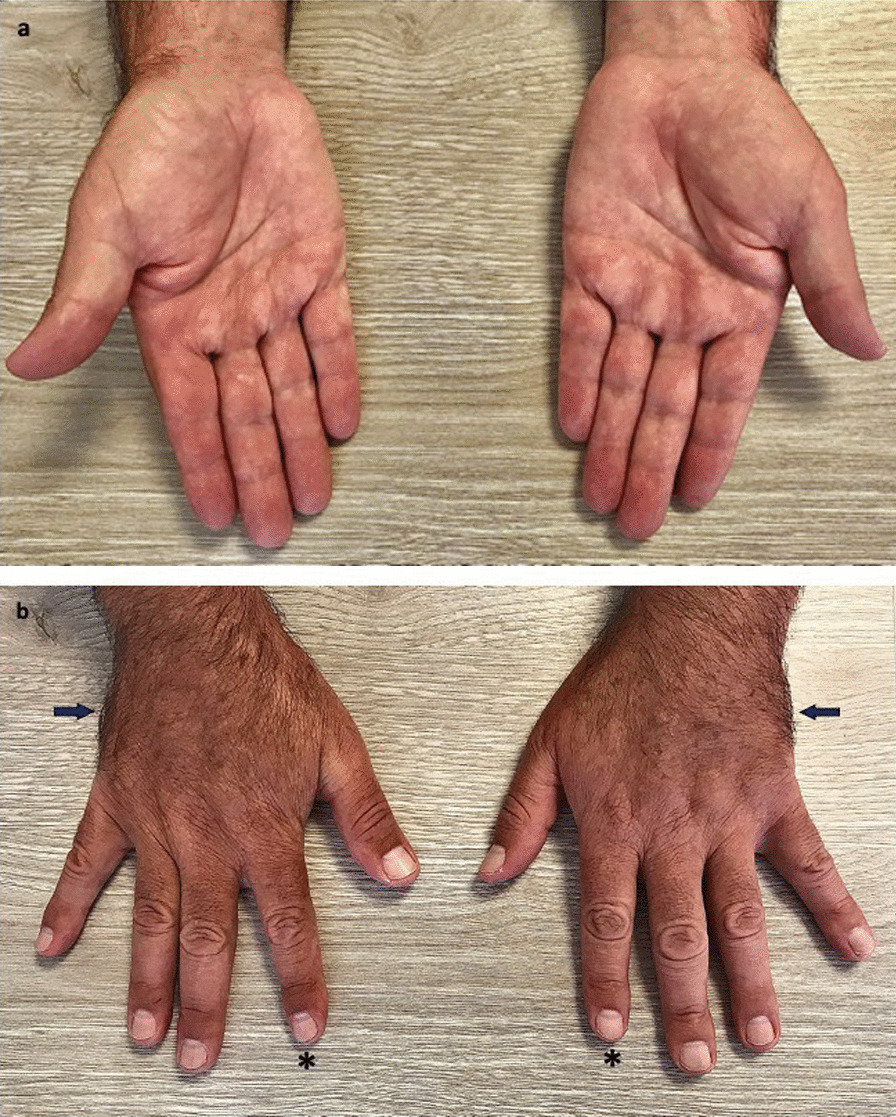
**a** Dusky and blue discolouration of fingertips with acrocyanosis. **b** White nails (asterisks) and hypertrichosis in the hands (arrows)

Twenty-four months later, the patient had paraparesis due to symmetric sensorimotor demyelinating polyneuropathy, and he was diagnosed with Guillain–Barre syndrome. Intravenous gammaglobulin (IVIg) was initiated at 0.4 g/kg/ day for five days, with an incomplete response. Raynaud’s phenomenon persisted, associated with acrocyanosis, hypertrichosis, and white nails (Fig. 1a, b). Additional laboratory test results were received, and ANA was positive but with a different pattern (1:1280 in a nucleolar pattern); serum protein electrophoresis revealed a polyclonal gammopathy with a monoclonal (M)-protein on immunofixation (IgG lambda), and increased concentrations of VEGF (827 pg/mL, reference value 31–86 pg/mL). 24-h urine protein measurement was 130 mg of protein per day (reference value: less than 150 mg per day), and a radiographic bone survey did not reveal sclerotic bone lesions. Another nailfold capillaroscopy was unremarkable, and tests for ENA and antiphospholipid antibodies were normal.

The Rheumatology staff proposed amyloidosis as a differential diagnosis due to the identification of monoclonal gammopathy and peripheral neuropathy. However, the patient did not have autonomic neuropathy, which may be present in 15% of patients with AL amyloidosis. Entrapment neuropathies such as carpal tunnel syndrome are frequent, but this manifestation was absent in our patient. Skin manifestations in amyloidosis are periorbital purpura, waxy thickening, macroglossia, and subcutaneous nodules. Nevertheless, a few cases with Raynaud’s syndrome have been described [6].

Another differential diagnosis proposed was type I cryoglobulinemia which frequently can occur with Raynaud*’*s phenomenon and peripheral neuropathy. In our case, the patient had demyelinating polyneuropathy compared to cryoglobulinemia, which occurs with sensory-motor axonal polyneuropathy. Other peripheral nervous system manifestations of cryoglobulinemia include small-fiber polyneuropathy and mononeuritis multiplex [7].

Based on the initial presence of superficial venous thrombosis, thrombocytosis, Raynaud’s phenomenon, hypertrichosis, white nails, acrocyanosis, and with the onset of demyelinating peripheral neuropathy, monoclonal gammopathy, and elevated levels of VEGF, the patient was diagnosed with POEMS syndrome by the Hematology group.

**Fig. 2:**
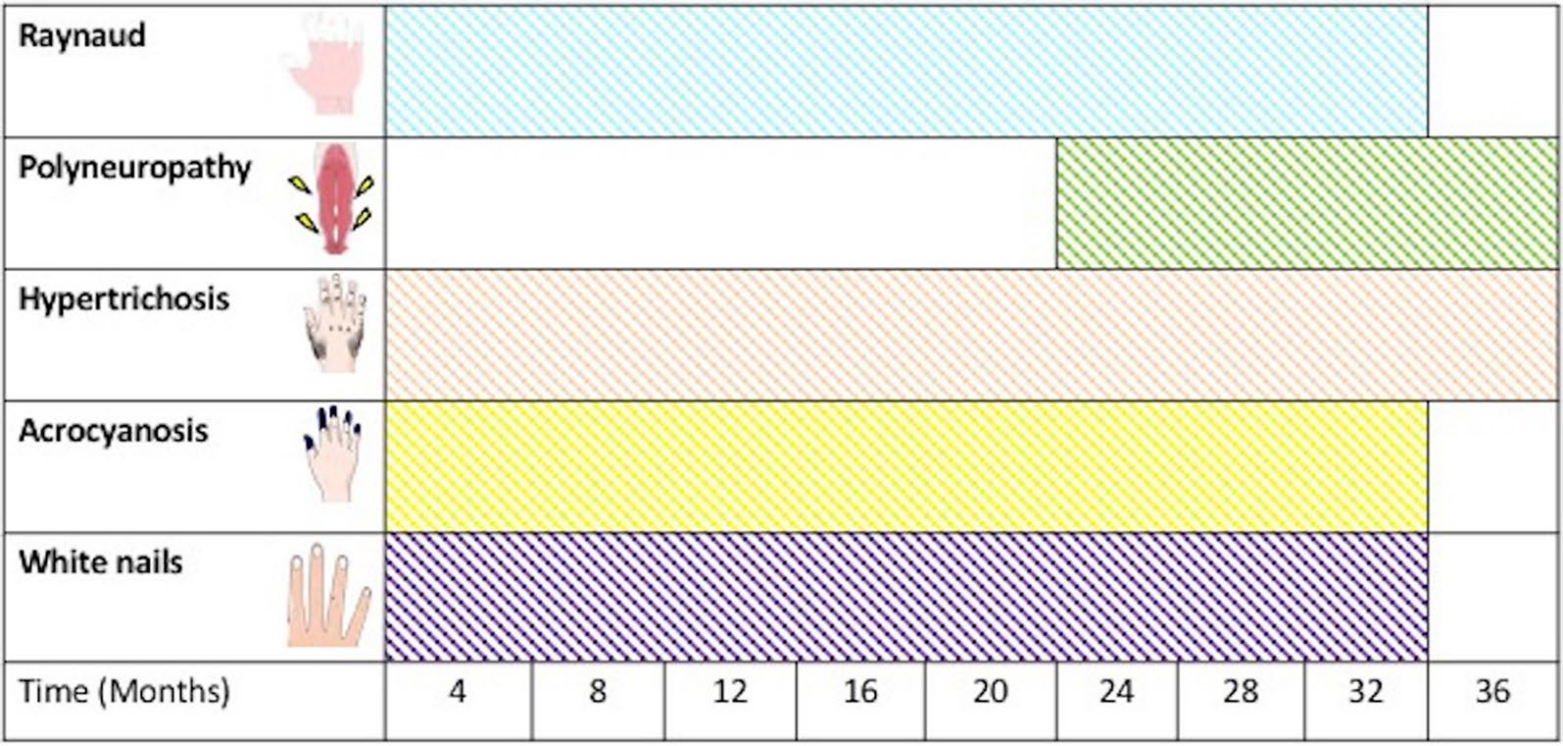
Timeline of the clinical signs in the case report

The bone marrow showed megakaryocytic hyperplasia and increased atypical plasma cells by 5 percent. Treatment with lenalidomide and dexamethasone was initiated, with the improvement of Raynaud’s phenomenon, acrocyanosis, and white nails. The patient showed neurologic partial response after 6 months and required a walker brace and physical therapy for mobility (Fig. 2). Autologous hematopoietic cell transplantation was proposed, pending authorization by the patient.

## Discussion and conclusions

POEMS syndrome presents with skin changes in 68–90 percent of patients, but Raynaud’s phenomenon is frequently overlooked [2]. It is present in twenty percent of patients and could be the first manifestation of this disease. The search for other skin lesions such as hemangiomas, hyperpigmentation, hypertrichosis, white nails, acrocyanosis, sclerodermoid changes, and hyperemia is important in the differential diagnosis of Raynaud’s phenomenon secondary to POEMS syndrome [3]. In our patient, the initial manifestations in the skin (minor criteria for the diagnosis) were followed by the presence of mandatory criteria (polyneuropathy and monoclonal gammopathy) and major criteria (elevated VEGF levels). Hence, this case highlights how some simple clinical findings can lead to a diagnosis of a complex syndrome.

Only one study describes the prevalence of Raynaud’s phenomenon in POEMS syndrome (Table 1) [5]. Sclerodermoid changes and skin localized thickening on the fingers that mimic SSc are described in 26–77% and 16%, respectively [4, 5, 8, 9]. These manifestations can be explained by the elevated concentrations of VEGF producing increased vascular permeability and proliferation with microangiopathy, capillary leakage, and vasoconstriction of arterioles and digital arteries [5]. Elevated VEGF levels also have been reported in patients with SSc, which could explain some of the similarities of skin findings between these diseases [10]. However, in contrast to POEMS syndrome, skin changes in SSc are due to the imbalance in turnover of extracellular matrix (EMC). The dysfunction in different factors and mechanisms involved in the repair of connective tissue after injury (endothelium, blood-derived cells) results in the activation of fibroblasts, increase in the deposit of fibronectin and type I collagen, and as a result the excessive synthesis of extracellular matrix [11, 12].

In addition, serum electrophoresis and immunofixation showed lambda-restricted monoclonal gammopathy [4], but the literature describes few cases with POEMS syndrome and the presence of autoantibodies. Only one case series reported a high prevalence of positive antimitochondrial antibody (AMA) and ANCA; notably, this study reported a higher prevalence of Raynaud’s phenomenon (31%) than the study of Mayo Clinic but not mentioned the presence of ANA [13].

In conclusion, the patients with Raynaud’s phenomenon associated with polyneuropathy, monoclonal gammopathy, or typical skin changes should be promptly investigated for POEMS syndrome. Our report describes the undocumented association between POEMS syndrome and positive ANA; we hypothesize that increased levels of cytokines (IL-1 β, TNF-α, and IL-6) activate clonal and polyclonal plasma cells with subsequent production of autoantibodies. The importance of this finding is unknown and possibly represents an epiphenomenon associated with the disease.

## Data Availability

Not applicable.

## Abbreviations

POEMS: Polyneuropathy, organomegaly, endocrinopathy, monoclonal gammopathy, and skin changes
ANA: Antinuclear antibodies
SPEP: Serum protein electrophoresis
VEGF: Vascular endothelial growth factor
SSc: Systemic sclerosis
IL-1β: Interleukin-1 beta
TNF-α: Tumor necrosis factor alpha
IL-6: Interleukin-6
TSH: Thyroid-stimulating hormone
ENA: Extractable nuclear antigen
ANCA: Antineutrophil cytoplasmic antibody
IVIg: Intravenous gammaglobulin
AMA: Antimitochondrial antibody
